# Fine Particulate Matter Concentrations during Independence Day Fireworks Display in the Lower Rio Grande Valley Region, South Texas, USA

**DOI:** 10.1155/2022/8413574

**Published:** 2022-09-12

**Authors:** Esmeralda Mendez, Owen Temby, Dawid Wladyka, Katarzyna Sepielak, Amit U. Raysoni

**Affiliations:** ^1^School of Earth, Environmental, and Marine Sciences, The University of Texas Rio Grande Valley, Brownsville, TX, USA; ^2^Department of Sociology, The University of Texas Rio Grande Valley, Brownsville, TX, USA

## Abstract

Fireworks are typically discharged as a mark of celebration and joy in many societies spanning various cultures. In the United States of America, 4^th^ July is celebrated as the Independence Day when the nation overthrew the British colonial yoke in 1776. While this day instills a sense of patriotism in every American's heart, it is also a major PM_2.5_ air pollution concern. This study is first of its type in the Lower Rio Grande Valley (RGV) Region of South Texas, USA, that characterizes fine particulate matter pollution. Using a low-cost sensor (TSI BlueSky Air Quality Monitor), real-time PM_2.5_ measurements were assessed at eleven different locations in four different towns and cities of Lower RGV Region: Brownsville, Edinburg, Weslaco, and Port Isabel. Hourly PM_2.5_ concentrations from July 03–06, 2021 are presented in this research work. Intraurban PM_2.5_ spatial and temporal variations provide an insight on the general population's exposure burden during the festive period. Results indicate an increase in fine particulate matter pollution across the region, but the levels do not exceed the U.S. National Ambient Air Quality Standards (NAAQS). Findings from this study would possibly help in the formulation of effective firework policies to minimize the pollution impact.

## 1. Introduction

Fireworks display can be a major source of air pollution and has the potential to cause deleterious health effects [1–4]. Of all the major criteria air pollutants, fine particulate matter (aerosol particles with an aerodynamic diameter of less than 2.5 *μ*m), i.e., PM_2.5_ is measured and studied due to its documented health effects [5–7]. PM_2.5_ particles can deposit deep in the lungs thereby resulting in damage to the lower thoracic region [8]. PM_2.5_ exposures have both acute and chronic impacts on human health [2, 3, 9–11]. Continued exposure to fine PM results in the development of relevant cardiovascular and or respiratory diseases [1, 4, 10]. The major sources of fine particulate matter pollution are typically traffic emissions including both tailpipe and break and tire tear and wear, power plant emissions, emissions from smokestacks, and agricultural burning [4, 12, 13].

Firework displays generate harmful concentrations of particulate matter and sulfur dioxide [4, 13–18]. Statistically significant impact between trace metals in PM_2.5_ from firework emissions on human health via reactive oxygen species formations has been well-documented [14, 19]. The assembly of a firecracker consists of multiple inorganic and organic chemicals such as sodium oxalate, strontium nitrate, barium nitrate, potassium perchlorate, potassium nitrates, arsenic, aluminum, charcoal, sulfur, manganese, and iron dust powder [11, 13, 14, 16, 17]. Explosion of fireworks also result in local haze [4].

A study published in 2015 reported that PM_2.5_ concentrations are elevated on 4^th^ July and continue to remain high until the morning of 5^th^ July [20]. The authors analyzed PM_2.5_ data from 315 US air quality monitory sites and showed a 42% increase (5 *µ*g/m³) of 24 hr PM_2.5_ concentrations in the United States during Independence Day [4, 20]. During the Chinese Lantern Festival in Beijing, Wang and colleagues showed a six-fold increase in PM_2.5_ concentrations on the Lantern Day as compared to normal days [17]. Another study from Beijing, China quantified PM_2.5_ average concentrations and documented highest levels during firework days at 248.9 *µ*g·m^−3^ in the 2015 Spring Festival. [4, 21, 22]. During the Montreal International Fireworks Competition in Quebec, Canada, researchers showed that PM_2.5_ levels can be as high as 1000 *µ*g/m³ during the display period of about 45 minutes [23]. In New Delhi, India, during the day of the Diwali (Festival of Lights) festival, PM_2.5_ levels were 588 *µ*g/m³ in 2007, and 389 *µ*g/m³ in 2008 [13].

Till date, no study has been conducted till date in the Lower RGV Region of South Texas, USA, to assess an increase in fine particulate matter pollution levels during the US Independence Day celebrations. The present study is first of its kind to use TSI BlueSky low-cost sensor to characterize fine particulate matter pollution in this region. Recently, the usage of low-cost air quality sensors to measure PM pollution has been gaining credence [9, 24]. These sensors are easy to operate, very convenient in terms of price, energy, and mobility, and record data in real time and can store data for future downloading purposes [9, 25].

## 2. Materials and Methods

### 2.1. Site Selection and Study Period

**Figure 1:**
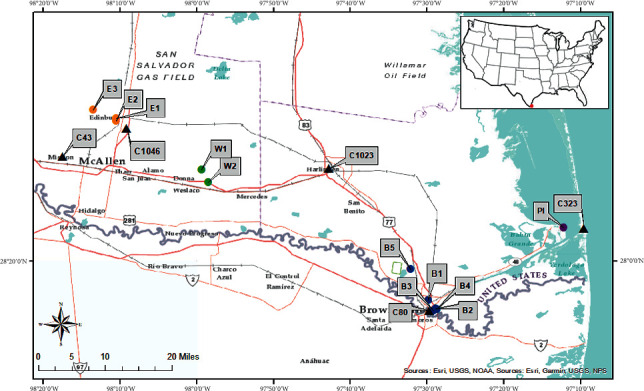
Map of Texas commission on environmental quality (TCEQ). Continuous ambient monitoring station (CAMS) sites and BlueSky air quality monitors throughout the study area in the lower RGV region.

The study area is in the Lower RGV Region of South Texas, the USA, with a population of 1,402,512 persons [26]. Eleven BlueSky sensors were deployed in four towns and cities and labeled as follows: five monitors in Brownsville (B1–B5), three monitors in Edinburg (E1–E3), two monitors in Weslaco (W1 and W2), and one monitor in Port Isabel (PI). These intraurban sensor locations are illustrated in Figure 1 with color-coordinated indicators to differentiate the cities. Brownsville locations are marked with blue indicators, Edinburg has orange, Weslaco has green, and lastly, Port Isabel is purple. Each marker signifies a deployed BlueSky monitor with tactical outdoor locations to portray significant areas such as schools, residential area, and other neighborhood locations.

Sensor B1 in Brownsville is right across from Dean Porter Park and adjacent to the Gladys Porter Zoo in a semiresidential area. B2 is deployed in the University of Texas Rio Grande Valley (UTRGV) Brownsville Campus police department, near the campus student dormitories. B3 is located at the Music and Science Learning Center on the University Boulevard at the UTRGV campus, and B4 is situated adjacent to Texas State Highway 69E only 0.04 miles away from the U.S.-Mexico International Port of Entry. The entire university is located on the main highway 69E on the edge of the US-Mexican border, as seen in Figure 1, so exposure to traffic pollutants from cross-border vehicular traffic is a health concern. B5 is in a neighborhood surrounded by Resaca del Rancho Viejo. Resacas are ancient distributary channels of Rio Grande River (the natural international boundary between the United States and Mexico in the State of Texas) and are a unique geological feature of this area.

Figure 1 also shows the locations of the five Continuous Ambient Monitoring Station (CAMS) sites in the region. These sites are maintained by the Texas Commission on Environmental Quality (TCEQ) and they monitor air pollutant such as PM_2.5_ and meteorological parameters such as temperature, resultant wind speed, and solar radiation. The CAMS sites are shown as black triangular symbol in the study map. The five CAMS sites are: C323 (Port Isabel), C80 (Brownsville), C1023 (Harlingen), C1046 (Edinburg), and C43 (Mission). Out of the five CAMS sites, only three record data for PM_2.5_. These are CAMS sites C43, C80, and C323. The other two C1046 and C 1023 do not collect PM_2.5_ data. All five sites collect various meteorological parameters such as temperature, resultant wind speed, and two sites (C43 and C80) provide data on solar radiation.

The UTRGV Edinburg campus has two monitors, E1 and E2 facing commonly used roads (Schunior St. and 107 Texas, respectively). E3 is deployed in a closed gated residential community. In Weslaco, W1 is located further from the city in a residence right off Farm to Market Road 88 (FM88). W2 is deployed at one of the buildings of the Weslaco Police department on the frontage of Texas State Expressway 83. Lastly, the PI sensor is deployed at the UTRGV coastal labs between two neighborhoods near the popular tourist destination of South Padre Island.

Sensor deployment locations were considered such that they are an accurate representation of the daily exposure levels of PM_2.5_ in the neighborhood. The RGV Region has previously hosted multiple fireworks in their cities, as well as supporting many fireworks stands, so residents can easily purchase firecrackers for their celebrations. In 2021, the cities and towns of Brownsville, Edinburg, Harlingen, McAllen, Weslaco, and South Padre Island celebrated the Independence Day with much fanfare by grand firework display shows.

The study period starts the evening of July 3^rd^, 7 : 00 pm through July 6^th^ at 6 : 59 pm. BlueSky monitors were configured to log PM concentrations every 5 minutes, and these data were collected by a built-in microSD card. Those 5-minute concentrations were formulated into hourly data to be further assessed. The day prior and after 4^th^ July are considered primarily as control days to help understand the temporal variation in the PM_2.5_ concentrations.

### 2.2. Instrumentation

In this study, BlueSky Low-Cost Sensors were used (Model: 8143 by TSI Incorporated, Minnesota, U.S.). This instrumentation is easy to install and weighs about 0.35 lb. The sensor does not require much power, approximately less than 5 W (5 VDC @ 1 Amp). The PM sensor included is precalibrated similarly to other high-quality TSI equipment like the DustTrak™ models (TSI Incorporated, Minnesota, U.S). Self-diagnostic tests are configured to daily cleaning intervals to attain high-quality data. The PM sensor measures from the range 0 to 1000 *μ*g/m^3^ with the measurement resolution of 1 *μ*g/m^3^ and a response time being 1 second [27]. It is prudent to mention here that, albeit the usage of low-cost sensors for air quality monitoring has increased substantially in the last few years, issues such as sensor baseline drift, sensitivity to variations in meteorological parameters such as ambient temperature and relative humidity, instrument measurement artifact needs to be addressed and accounted for as outlined succinctly by Morawska and colleagues [28]. Extensive quality assurance and quality control (QA and QC) studies conducted by the South Coast Air Quality Management District (South Coast AQMD) on BlueSky sensors have shown a good record of performance and evaluations with the sensor showing a moderate to strong PM_2.5_ (0.66 < *R*^2^ < 0.78) correlation with other Federal Equivalent Method (FEM) instruments like FEM GRIMM and FEM Teledyne API T640 in the field [29].

### 2.3. Statistical Data Analysis

Data from these low-cost sensors were inputted in Microsoft Excel (2021) for calculating hourly concentrations and time series. The time series depicts PM_2.5_ concentrations through the study period hours, while also comparing data from each site. Spearman's Rho correlations were calculated with SPSS for MacOS (SPSS, Inc., Chicago, IL) to estimate temporal variability in PM_2.5_ concentrations across the eleven locations. Visual data analysis from R programming (RStudio, Inc., Boston, MA) was used to demonstrate box plot hourly variability. The boxes are the interquartile ranges (75^th^ and 25^th^), the whiskers show the minimum and maximum values, the outliers are shown in asterisk. The median is indicated by the black line inside the boxes and the diamond in the boxes indicate the mean.

Spatial variation in PM_2.5_ concentrations in each site are analyzed with the performance of Coefficient of Divergence (COD) analysis was performed to study the spatial variation between the various study sites [30–32]. The COD provides a degree of uniformity between two simultaneously sampled sites, *j* and *k* by the following equation:

(1) $$\begin{array}{c} {\text{COD}}_{jk}=\sqrt{\frac{1}{p}\sum_{i-1}^{p}{\left[\frac{x_{ij}-x_{i,k}}{x_{ij}+x_{i,k}}\right]}^{2}}, \end{array}$$

where the number of observations is indicated by *p* and *x*_*ij*_ is the *i*^th^ concentration measured at site *j* over the sampling period. COD values less than 0.20 indicate the two observed sites are spatially homogeneous in terms of the pollutant concentration. COD equal to greater than 0.20 establishes spatial heterogeneity in the pollutant concentrations or significant differences between the two simultaneously sampled sites [31].

## 3. Results and Discussion

### 3.1. PM_2.5_ Concentrations

**Figure 2:**
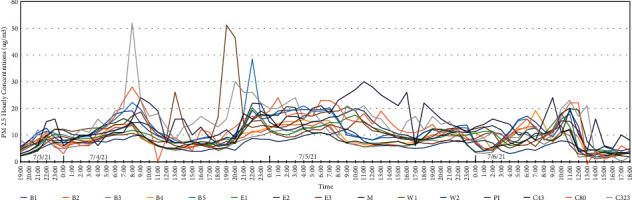
Time series of PM_2.5_ concentrations (*µ*g/m³) at the study locations and the CAMS sites.

Time series was plotted for the data available from the evening of June 03, 2021 (19 : 00 hours) till the evening of July 06, 2021, (19 : 00 hours) as shown in Figure 2. Hourly basic statistics for PM_2.5_ for the entire duration are shown in Table 1 and the meteorological parameters are shown in Table 2. The weather conditions during the study period were stable with the mean temperature in the region around 30°C. The resultant wind speed also ranged from 1.97 to 3.36 m/s across the five CAMS sites. The time series shows the average hourly data PM_2.5_ in *µ*g/m³. The time series expressions are estimated as hourly averages to better interpret any easily identifying variations. The four-day duration is labeled accordingly on the *x* axis. An evident increase in spike starting the evening of July 4^th^ at approximately 20 : 00 hours is obvious with high concentrations lingering till the morning hours of July 5. The sensor W1, a location in the town of Weslaco, recorded the highest hourly PM_2.5_ concentrations (17.2 ± 10.6 *µ*g/m³) compared to the other locations from July 04, 19 : 00 hours to July 05, 18 : 59 hours. In contrast, W2 site recorded the lowest PM_2.5_ concentrations (7.9 ± 2.3 *µ*g/m³) for the same time frame.

In the city of Brownsville, across the five sampling sites one can observe a slight increase in mean PM_2.5_ concentrations on the day of Independence. For example, site B1 showed the mean concentration of 13.6 ± 4.9 *µ*g/m³ for between July 04, 19 : 00 hours- July 05, 18 : 59 hours. For the same time frame, site B3 had the lowest concentration (8.8 ± 2.9) *µ*g/m³ and site B5 had the highest (14.8 ± 7.1) *µ*g/m³. In the city of Edinburg, hourly concentrations ranged from 11.1 ± 2.7 *µ*g/m³ at E3 to 14.1 ± 4.1 *µ*g/m³ at E2 for the same study period. Similarly, the sensor at Port Isabel (PI) showed a mean of 10.8 ± 4.7 *µ*g/m³. The PM_2.5_ concentrations reverted to control values starting July 05. As is obvious from Tables 1 and 2, the mean concentrations varied from the lowest at W2 (5.3 ± 2.1 *µ*g/m³) to the highest at B1 (9.7 ± 5.1 *µ*g/m³) and E2 (9.7 ± 4.1 *µ*g/m³). It is also important to mention here a striking similarity in the pollutant concentrations a day prior and after the 4^th^ of July Independence Day celebrations as is obvious from Table 1.

Site W1 is located in a residential area as mentioned before and it is quite probable that the huge amount of fireworks lit in this neighborhood by the residents resulted in an hourly maximum of 51.2 *µ*g/m³ on the Independence Day. For that same period, the maximum recorded at site W2 is only 12.3 *µ*g/m³. This makes sense because site W2 is located at one of the buildings of the Weslaco Police Department and there were no fireworks reported in this neighborhood. This same trend is observed in the city of Brownsville. Sites B1 and B5 were in residential neighborhoods where we expect people to light firecrackers. Hence the maximum concentration recorded at these two sites were 20.3 *µ*g/m³ (B1) and 38.5 *µ*g/m³ (B5). This is in sharp contrast to site B4 (adjacent to the interstate highway) where the maximum concentration was recorded as 15.5 *µ*g/m³, as well as site B3 which is the University campus. These findings, therefore, suggest the role of fireworks in the high pollutant concentration observed during this festive period.

The three CAMS sites also exhibited higher PM_2.5_ concentrations between July 04 and July 05. The mean concentration at C43 (Mission) was 19.54 ± 5.96 *µ*g/m³, C80 (Brownsville), 16.54 ± 3.82 *µ*g/m³, and C1046 (Edinburg), 18.17 ± 5.38 *µ*g/m³. The maximum hourly concentration recorded at C43 and C1046 for this period is 30 *µ*g/m³. For the next 24-hour period, the PM_2.5_ values at the three CAMS sites ranged from 9.67 ± 6.03 *µ*g/m³ (C80) to 12.08 ± 4.83 *µ*g/m³ (C43) suggesting a decrement in the pollutant concentrations after the firework festive period. These values between July 05 and July 06 (post celebrations) reflect the same low concentration trend as was observed between July 03 and July 04 (pre celebrations, 10.78 ± 6.48 *µ*g/m³ at C80 to 14.83 ± 9.02 *µ*g/m³ at C1046).

The United States Environmental Protection Agency is mandated by the United States Congress, under the auspices of the Clean Air Act of 1970 and its amendments thereof in 1990 and onwards, to set National Ambient Air Quality Standards (NAAQS) for the criteria air pollutants-one of which is PM_2.5._ Currently, the 24-hour mean average for PM_2.5_ is 35 *µ*g/m³. Based on our research findings, we can posit that none of the study sites during the three-day study period exceeded the PM_2.5_ NAAQS standards. However, it would be prudent to mention here that the daily mean PM_2.5_ standard by the World Health Organization is 15 *µ*g/m³ and our study sites are just within this stricter standard as well during the study period.

**Figure 3:**
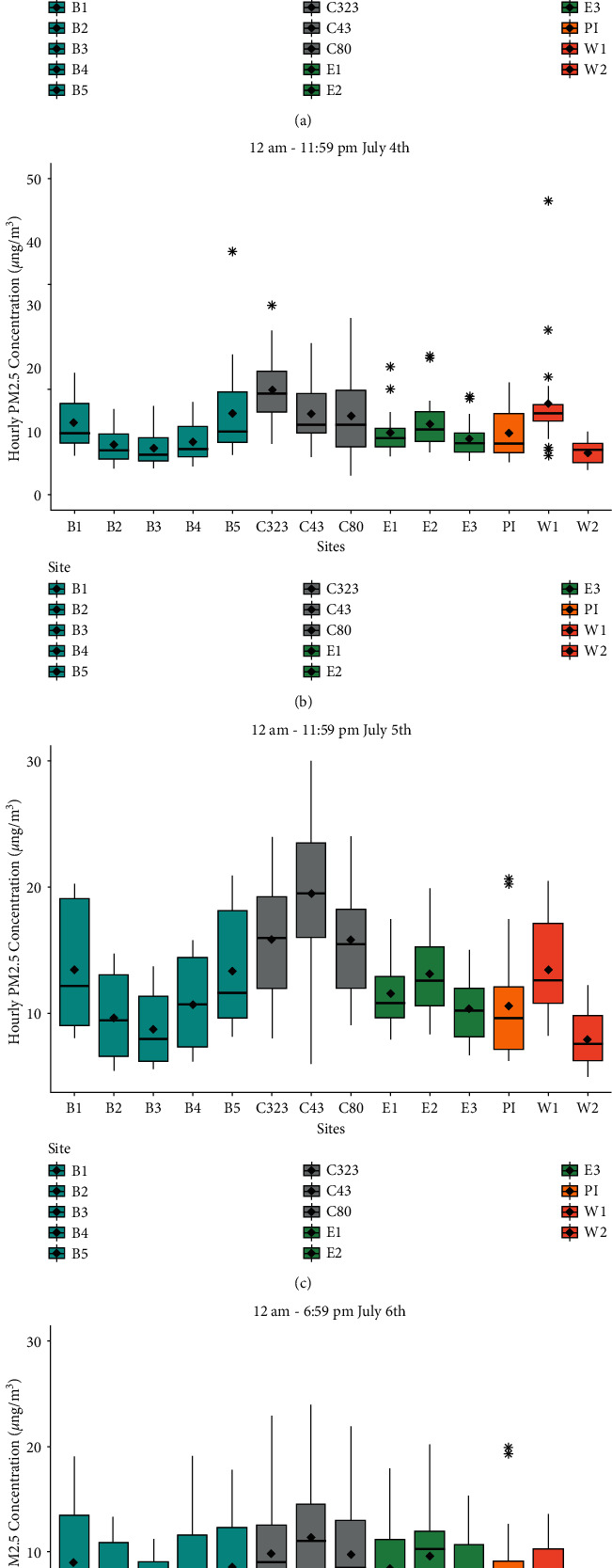
Box plots of hourly average concentrations of PM_2.5_ (µg/m³) from the study locations and the three CAMS sites.

Figure 3 displays the box plots for the hourly PM_2.5_ concentrations across the eleven sites and the three CAMS sites from July 03 to July 06. The color coordination for the box plots is as followed: blue for Brownsville, gray for the CAMS sites, green for Edinburg, gold for Port Isabel, and salmon for Weslaco. Box plots are helpful to detect any noticeable outliers, which are seen during for PM_2.5_ concentrations on the Independence Day. The high values on this day and the outliers accentuate the role play by firework displays across most of the sites. Outliers are also observed for July 05 and July 06 for Port Isabel, close to South Padre Island-a major U.S. tourist destination that organizes massive firework displays.

### 3.2. Spatial and Temporal Variations in PM_2.5_ Hourly Concentrations

COD matrix for PM_2.5_ concentrations across the eleven sites and the three CAMS sites are shown in Table 3. COD values equal to greater than 0.2 are in italic and bold. In Brownsville, spatial uniformity in PM_2.5_ concentrations are observed by values less than 0.2 for the following pairings: B1–B4 (0.15), B1–B5 (0.15), B2–B3 (0.1), B2–B4 (0.15), and B2–B5 (0.19). Values are equal to and greater than 0.2 suggest a slight spatial heterogeneity between sites B1–B2 (0.21), B1–B3 (0.26), B3–B4 (0.2), B3–B5 (0.23), and B4–B5 (0.21). Spatial variation is more pronounced between the CAMS site in Brownsville (C80) and the five Brownsville study sites. B1 (0.32), B2 (0.45), B3 (0.47), B4 (0.37), and B5 (0.42). These COD values suggest that central ambient sites such as TCEQ CAMS may not be an accurate representation of the actual PM_2.5_ exposure burden in any urban setting. The two sampling sites at Weslaco exhibit a COD value 0.3 indicating spatial nonuniformity. In the city of Edinburg, spatial homogeneity was observed across the three sampled sites with COD values less than 0.2. However, the three Edinburg sites demonstrated spatial nonuniformity in the PM_2.5_ concentrations when compared with the CAMS C43 site in Mission (E1: 0.27, E2: 0.23, and E3: 0.30). Port Isabel sampling site also exhibited spatial heterogeneity in PM_2.5_ pollutant concentrations with all the sampled sites except for B1 (0.19) and B4 (0.17). The COD value (0.32) between PI site and C323 site in Port Isabel also exhibit slight spatial nonhomogeneity. These COD findings demonstrate the importance of intra- and interurban sampling of criteria air pollutants like fine particulate matter.

Spearman's rho correlation coefficients were computed between all the study and CAMS sites for fine particulate matter and are shown in Table 4. The correlation coefficients were all statistically significant at the 0.01 level. A stronger relationship across two simultaneously sample sites would have a higher value, whereas a weaker relationship would indicate a lower coefficient. All the site pairings yielded a statistically significant and positive correlations. Across the city of Brownsville, all the sites were very strongly correlated with *r* > 0.8. Similarly, the three Edinburg sites were very robustly correlated with *r* > 0.9. On similar lines, sites W1 and W2 were correlated with each other (*r* = 0.77). The Port Isabel site was positively correlated with all the sites exhibiting a very strong relationship with site (B1, *r* = 0.84) and weakly correlated with site W1 (*r* = 0.35). The CAMS site C80 was also very strongly correlated with all the study locations (0.506 < *r* < 0.820). C323 was, however, weakly correlated with C43 (*r* = 0.292). All the study sites were weakly to moderately correlated with C43 (0.233 < *r* < 0.640) except for the Port Isabel study location (*r* = 0.178). The correlation coefficient values suggest that across the lower RGV region landscape ubiquity in temporal increase or decrease of PM_2.5_ concentrations is observed.

## 4. Conclusions

To the best of our knowledge, the findings from this study are the first in the lower RGV region of South Texas, characterizing fine particulate matter pollution across eleven sites before, during, and after the 4^th^ of July Independence Day celebrations. These celebrations are accompanied by the display of massive fireworks display resulting in short-term increase in particulate matter pollution. Whereas, our study findings do not exceed the US NAAQS 24-hr mean PM_2.5_ concentrations, even a short-term exposure of a few hours to high PM_2.5_ levels can result in respiratory health issues such a wheezing, tightness of chest, and asthma aggravation and subsequent asthma attacks. Findings from this study may, therefore, contribute toward the future formulation of policies for firework display at the local level. This study demonstrates the usefulness of using low-cost sensors to characterize intraurban spatial variability in fine particulate matter concentrations as well as address fine-scale changes in PM_2.5_ pollution, which is inadequately captured by federal and state fixed continuous ambient monitoring sites.

## Figures and Tables

**Table 1:** Basic statistics of hourly PM_2.5_ concentrations during the study period at the various study locations and the CAMS sites.

Date	Sites	B1	B2	B3	B4	B5	E1	E2	E3	W1	W2	*P*I	C43	C80	C1046
July 03, 19 : 00—July 04, 18 : 59 hours	M	10.6	7.5	7.0	8.0	10.9	8.4	9.5	7.4	11.9	6.2	8.4	11.83	10.78	14.83
	STD	3.9	2.7	2.7	3.0	4.4	2.3	3.1	2.2	4.8	2.0	3.8	5.16	6.48	9.02
	MAX	19.1	13.6	14.0	14.6	22.2	11.8	14.8	10.7	26.1	9.9	16.2	24.00	28.00	52.00
	MIN	5.7	3.8	3.7	4.5	4.7	3.0	3.0	2.3	4.4	2.2	2.2	5.00	3.00	6.00
	Median	9.4	6.9	6.1	7.1	9.7	8.8	10.0	7.5	12.2	6.5	7.7	5.5	6	7.4

July 04, 19 : 00—July 05, 18 : 59 hours	M	13.6	9.6	8.8	10.4	14.8	12.5	14.1	11.1	17.2	7.9	10.8	19.54	16.54	18.17
	STD	4.9	3.5	2.9	3.6	7.1	3.2	4.1	2.7	10.6	2.3	4.7	5.96	3.82	5.38
	MAX	20.3	14.5	13.9	15.7	38.5	18.8	21.9	15.5	51.2	12.3	20.7	30.00	24.00	30.00
	MIN	6.7	4.9	5.1	5.5	6.3	7.3	8.3	6.8	8.3	4.3	6.3	6.00	11.00	9.00
	Median	11.8	8.9	8.3	9.7	15.2	12.1	13.9	11.3	14.2	7.8	8.6	6.65	7.7	6.9

July 05, 19 : 00—July 06, 18 : 59 hours	M	9.7	7.5	6.4	8.6	9.4	8.8	9.7	8.0	7.6	5.3	8.3	12.08	9.67	10.50
	STD	5.1	3.9	3.2	4.5	4.8	3.7	4.1	3.6	4.3	2.1	4.9	4.83	6.03	6.35
	MAX	19.1	15.3	10.8	19.3	18.2	17.7	20.1	15.3	13.8	8.0	19.9	24.00	22.00	23.00
	MIN	2.6	1.9	1.9	2.3	2.0	3.4	4.1	2.8	1.2	1.8	1.9	4.00	0.00	0.00
	Median	11.5	8.5	7.1	9.9	10.5	9.2	10.3	7.8	7.5	5.5	7.8	4.4	6.9	7.9

**Table 2:** Meteorological parameters during the study period at the various CAMS sites.

Met parameters	CAMS sites	*July 03, 19* : *00—July 04, 18* : *59 hours*				*July 04, 19* : *00—July 05, 18* : *59 hours*				*July 05, 19* : *00—July 06, 18* : *59 hours*			
		Mean	STD	Min	Max	Mean	STD	Min	Max	Mean	STD	Min	Max
Temp (°C)	C43	30.06	3.59	25.44	35.72	29.28	2.62	26.11	33.67	26.98	1.89	24.44	32.17
	C80	29.17	2.20	26.72	32.78	29.02	2.30	25.72	32.44	26.95	2.28	22.67	30.06
	C323	30.18	1.24	28.72	32.00	30.25	1.04	28.94	32.00	28.69	2.26	24.11	31.11
	C1023	29.51	2.67	26.33	33.89	29.37	2.71	26.06	33.44	26.58	2.36	22.39	31.11
	C1046	29.92	3.37	25.78	35.17	29.44	3.08	25.83	34.00	26.73	2.14	23.56	31.89

RWS (m/s)	C43	2.83	0.90	1.74	4.96	3.09	0.80	1.21	5.27	2.08	1.03	0.27	3.84
	C80	2.97	1.07	1.52	4.74	3.32	1.56	0.13	5.10	2.98	1.44	0.13	5.36
	C323	3.38	1.07	1.65	4.96	3.36	0.70	2.32	4.87	3.50	0.72	1.52	4.78
	C1023	3.02	1.09	1.52	5.23	3.66	1.17	1.70	5.54	2.73	0.81	1.52	4.34
	C1046	2.69	0.84	1.30	4.34	3.03	1.20	1.21	5.14	1.97	1.25	0.22	4.51

Solar	C43	0.43	0.52	0.00	1.35	0.26	0.31	0.00	0.87	0.04	0.06	0.00	0.19
Radiation (Langleys per minute)	C80	0.33	0.45	0.00	1.39	0.31	0.39	0.00	1.20	0.07	0.12	0.00	0.42

**Table 3:** PM_2.5_ COD values between the study locations and the CAMS sites.

PM_2.5_	B2	B3	B4	B5	E1	E2	E3	W1	W2	PI	C43	C80	C323
B1	0.21	0.26	0.15	0.15	0.23	0.19	0.22	0.26	0.3	0.19	0.29	0.32	0.27
B2		0.1	0.15	0.19	0.2	0.27	0.23	0.31	0.24	0.21	0.37	0.45	0.37
B3			0.2	0.23	0.24	0.31	0.25	0.33	0.23	0.23	0.40	0.47	0.40
B4				0.21	0.23	0.22	0.19	0.27	0.23	0.17	0.33	0.37	0.33
B5					0.19	0.22	0.26	0.28	0.33	0.24	0.30	0.42	0.30
E1						0.15	0.15	0.25	0.25	0.24	0.27	0.33	0.32
E2							0.13	0.23	0.28	0.23	0.23	0.28	0.27
E3								0.25	0.19	0.2	0.30	0.35	0.32
W1									0.3	0.31	0.29	0.40	0.29
W2										0.24	0.40	0.41	0.41
PI											0.36	0.29	0.32
C43												0.34	0.32
C80													0.28

**Table 4:** Correlation Coefficients for PM_2.5_ concentrations between the study locations and the CAMS sites.

	B1	B2	B3	B4	B5	E1	E2	E3	W1	W2	PI	C43	C80	C323
B1	1													
B2	0.923^*∗∗*^	1												
B3	0.922^*∗∗*^	0.955^*∗∗*^	1											
B4	0.919^*∗∗*^	0.937^*∗∗*^	0.861^*∗∗*^	1										
B5	0.940^*∗∗*^	0.915^*∗∗*^	0.934^*∗∗*^	0.844^*∗∗*^	1									
E1	0.522^*∗∗*^	0.519^*∗∗*^	0.524^*∗∗*^	0.531^*∗∗*^	0.606^*∗∗*^	1								
E2	0.564^*∗∗*^	0.496^*∗∗*^	0.512^*∗∗*^	0.521^*∗∗*^	0.601^*∗∗*^	0.954^*∗∗*^	1							
E3	0.527^*∗∗*^	0.437^*∗∗*^	0.451^*∗∗*^	0.500^*∗∗*^	0.510^*∗∗*^	0.915^*∗∗*^	0.952^*∗∗*^	1						
W1	0.416^*∗∗*^	0.384^*∗∗*^	0.424^*∗∗*^	0.409^*∗∗*^	0.478^*∗∗*^	0.652^*∗∗*^	0.637^*∗∗*^	0.557^*∗∗*^	1					
W2	0.639^*∗∗*^	0.572^*∗∗*^	0.583^*∗∗*^	0.627^*∗∗*^	0.667^*∗∗*^	0.867^*∗∗*^	0.874^*∗∗*^	0.795^*∗∗*^	0.772^*∗∗*^	1				
PI	0.839^*∗∗*^	0.777^*∗∗*^	0.743^*∗∗*^	0.803^*∗∗*^	0.767^*∗∗*^	0.485^*∗∗*^	0.525^*∗∗*^	0.522^*∗∗*^	0.352^*∗∗*^	0.602^*∗∗*^	1			
C43	0.275^*∗*^	0.246^*∗*^	0.292^*∗*^	0.233^*∗*^	0.361^*∗∗*^	0.640^*∗∗*^	0.607^*∗∗*^	0.557^*∗∗*^	0.474^*∗∗*^	0.519^*∗∗*^	00.178	1		
C80	0.820^*∗∗*^	0.701^*∗∗*^	0.752^*∗∗*^	0.716^*∗∗*^	0.784^*∗∗*^	0.638^*∗∗*^	0.693^*∗∗*^	0.686^*∗∗*^	0.523^*∗∗*^	0.661^*∗∗*^	0.704^*∗∗*^	0.506^*∗∗*^	1	
C323	0.510^*∗∗*^	0.411^*∗∗*^	0.455^*∗∗*^	0.399^*∗∗*^	0.519^*∗∗*^	0.463^*∗∗*^	0.547^*∗∗*^	0.508^*∗∗*^	0.487^*∗∗*^	0.501^*∗∗*^	0.601^*∗∗*^	0.292^*∗*^	0.680^*∗∗*^	1

^*∗∗*^ Correlation is significant at the 0.01 level (2-tailed), *N* = 71-72 for all pairs.

## Data Availability

The data used to support the findings of this study are available from the corresponding author upon request.
